# Supplementary material for: Early prediction of Alzheimer’s disease using longitudinal electronic health records of US military veterans
Source: Commun Med (Lond). 2026 Jan 12;6:23. doi: 10.1038/s43856-025-01206-w (PMC12796311; doi:10.1038/s43856-025-01206-w)
Supplement: Supplementary file 2 — Supplementary file [file 43856_2025_1206_MOESM2_ESM.pdf]

Supplementary information

Early Prediction of Alzheimer's Disease Leveraging Symptom Occurrences from Longitudinal Electronic Health Records of US Military Veterans

Supplementary Box 1. Expert-curated keywords for identifying SCD and AD-relevant signs and symptoms.
Supplementary Box 2. Dermatologic-related control keywords
Supplementary Note 1. Predictive Models and Implementation Methods
Supplementary Figure 1. Study timeline and prediction window setting.
Supplementary Figure 2. SCD and AD dementia-related keyword occurrences on different keyword groups in longitudinal EHRs.
Supplementary Figure 3. SCD and AD dementia -related keyword patterns in primary care notes.
Supplementary Figure 4. SCD and AD dementia-related keyword patterns in longitudinal EHRs stratified by age.
Supplementary Figure 5. SCD and AD dementia-related keyword patterns in longitudinal EHRs stratified by sex.
Supplementary Figure 6. SCD and AD dementia-related keyword patterns in longitudinal EHRs stratified by ethnicity/race.
Supplementary Figure 7. SCD and AD dementia-related keyword patterns in longitudinal EHRs stratified by symptom domain.
Supplementary Figure 8. SCD and AD dementia-related keyword patterns in longitudinal EHRs in the CP-II cohort.
Supplementary Figure 9. SCD and AD dementia-related keyword patterns in longitudinal EHRs stratified by note types.
Supplementary Figure 10. Distribution of note types by specialty in the case and control cohorts in CP-I cohort.
Supplementary Figure 11. SCD and AD dementia–related keyword counts by category over the 3 years preceding diagnosis.
Supplementary Figure 12. Note volume by type in the 3 years preceding diagnosis.
Supplementary Figure 13. Dermatologic-related keyword patterns in longitudinal EHRs.
Supplementary Figure 14. Accumulated keywords feature group importance ranking of random forest model.
Supplementary Figure 15. Keywords importance ranking of random forest model on CP-I.
Supplementary Figure 16. Keywords importance ranking of random forest model on CP-II.
Supplementary Figure 17. Keywords importance ranking of random forest -1-day model on female/male subgroups.
Supplementary Figure 18. Keywords importance ranking of random forest -1-day model on white and black/African subgroups.
Supplementary Figure 19. Keywords importance ranking of random forest -1-day model on Non-Hispanic/Latino and Hispanic/Latino subgroups.
Supplementary Table 1. AD-related ICD code description.
Supplementary Table 2. ICD Codes used to identify and exclude dementia patients from control groups.
Supplementary Table 3. AD-related stop codes within VHA.
Supplementary Table 4: Logistic regression and XGBoost prediction results using keyword features in Setting I on the CP-I cohort
Supplementary Table 5: Random forest prediction results using keyword features from different specialty note types in Setting I on the CP-I cohort.

This supplemental material has been provided by the authors to give readers additional information about their work.

Supplementary Box 1. Expert-curated keywords for identifying SCD and AD-relevant signs and symptoms.

Cognition

- **Speech/language**
  - communication, speech, speaking
  - word-finding/retrieval, naming, encoding, phonemic, aphasia, paraphasia, anomia, dysnomia
  - fluency, perseveration, repetition
  - language, linguistic
  - comprehend, understand, alexia
- **Memory**
  - memory, amnesia, amnestic
  - remembering, recognizing, recall recount, retain
  - forget, lapse
- **Learning/Perception**
  - attention, concentration, focus, learning, abstraction, problem-solving
  - executive function, cognitive, neurocognitive, thinking, processing
  - visuospatial, multidomain, global, agnosia
  - getting lost, trouble finding, disoriented, confusion
  - Handwriting deterioration

Assistance Needed

- ADLs (Activities of Daily Living), self-care, eating, dressing, grooming, toileting, bathing, mobility
- iADLs (Instrumental Activities of Daily Living), cooking, housekeeping, cleaning, laundry, shopping, phone use, computer use, managing medications, managing bills/finances, driving/transportation, medical and legal decision-making, healthcare proxy, HPOA (Healthcare Power of Attorney), guardian/guardianship, supervision required

Physiological Changes

- hearing, auditory, SNHL (sensorineural hearing loss), HoH (hard of hearing)
- vision
- smell, anosmia, hyposmia
- swallowing, dysphagia
- gait, balance
- sleep, insomnia
- pain
- incontinence

Neuropsychiatric Symptoms

- mood
- affect, behavior, apathy
- personality
- *depressed, anhedonia*
- *anxiety, anxious, agitation, hypervigilance, restless, overwhelmed*
- insight, judgment, *impulsive*, anosognosia
- *anger, short-tempered, irritable, aggressive, shouting*
- erratic, rummaging
- wandering
- thought disorder
- delusion, hallucination, paranoia, psychosis

Supplementary Box 2. Dermatologic-related control keywords

General Symptoms: rash, itching/pruritus, dry skin, flaky skin, redness, skin irritation, cracked skin, skin pain, bruising, swelling, tender skin, burning sensation, oozing skin, inflamed skin, blister, peeling skin

Localized Findings: skin lesion, skin ulcer/ulcer, skin lump/bump, sore, scab, abscess, skin tag, mole, wart

Diagnoses / Conditions: eczema, psoriasis, acne, hives, dermatitis, contact dermatitis, atopic dermatitis, cellulitis, fungal infection, ringworm (tinea), scabies, shingles (herpes zoster), folliculitis, skin infection

Supplementary Note 1. Predictive Models and Implementation Methods

Models

A random forest (RF) algorithm consists of many decision trees. The "forest" generated by the RF algorithm is trained through bagging or bootstrap aggregating, which helps improve the robustness of the models. The prediction is made by taking the mean of the output from various trees.

Logistic Regression (LR) formulates the probability of one event as the log-odds of a linear combination of one or more independent variables (predictors) and learns to assign weights to different variables for making predictions.

Extreme Gradient Boosting (XGBoost) is an optimized implementation of gradient boosting that builds an ensemble of decision trees in a sequential manner. Each new tree is trained to correct the residual errors made by the previous trees, gradually improving the model's accuracy.

Parameters

All the models are implemented using Scikit-learn. The parameters are set as follows:

RF Hyper-parameters were tuned (grid search) on the 10% model tuning set to determine parameters of n_estimators, max_depth (3, 5, 7, 9), bootstrap (true, false), min_samples_leaf (1, 2, 4), min_samples_split (2, 5, 10) and max_features (sqrt, auto). The number of estimators and the maximum depth were adjusted to strike a balance between performance and overfitting. Additionally, a subset of features (max_features) was used for each tree to mitigate the impact of high feature correlation.

Logistic Regression: Penalty l2, C=1.0, intercept_scaling=1, max_iter 1000

XGBoost: max_depth=10, learning_rate=0.1, n_estimators=100, subsample=0.8, colsample_bytree=0.8, objective='binary:logistic', random_state=0

TF-IDF

The following equation depicts how the TF-IDF values of a specific keyword represented as W_i are calculated. tf_{ip} is the frequency of keyword k_i in a specific patient $patient_p$'s records. N is the total number of patients in the considered group (training data) and df_i is the total number of patients that have k_i in their records.

$$W_i^j = tf_{ip}^j \times log(\frac{N}{df_i})$$

Structured data features

we extracted the ICD diagnosis codes, medication, and abnormal measurements before the index time for the AD cases and controls. ICD codes are processed similarly as keywords using TF-IDF, and medications are mapped to the VA drug classes including 576 classes.

Top feature interpretation

RF models were explored for feature interpretation. The average decrease in Gini impurity for each feature was used to assess feature importance in the RF models. This importance was averaged across different time points (-10 years, -7 years, -5 years, -3 years, -1 year, and -1 day) to determine the overall importance for each model type. These values were then normalized by the maximum importance value across all time points within each model type. Finally, feature importances were ranked within each model to determine their relative importance at each time point.

Supplementary Figure 1. Study timeline and prediction window setting.

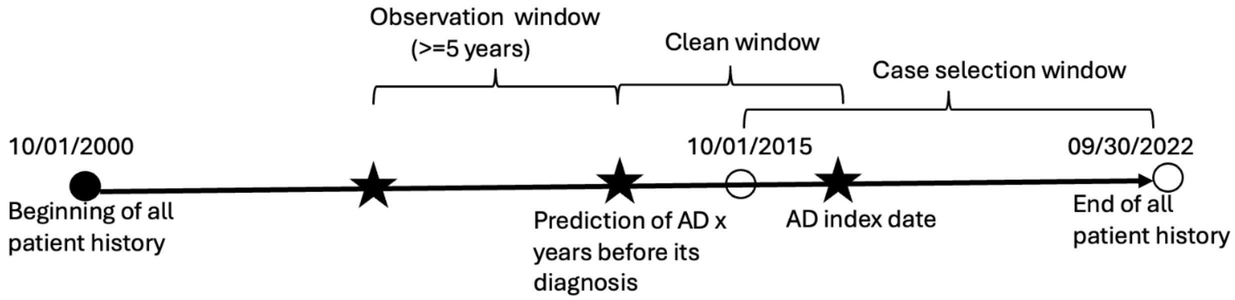

The study period is from 10/1/2000 to 09/30/2022 and AD index date is after 10/1/2015. At least 5 years of longitudinal EHRs are used at the time of prediction. Clean period during which no data are used in predicting AD development could vary from 0 to 10 years. AD, Alzheimer’s disease.

Supplementary Figure 2. SCD and AD dementia-related keyword occurrences on different keyword groups in longitudinal EHRs.

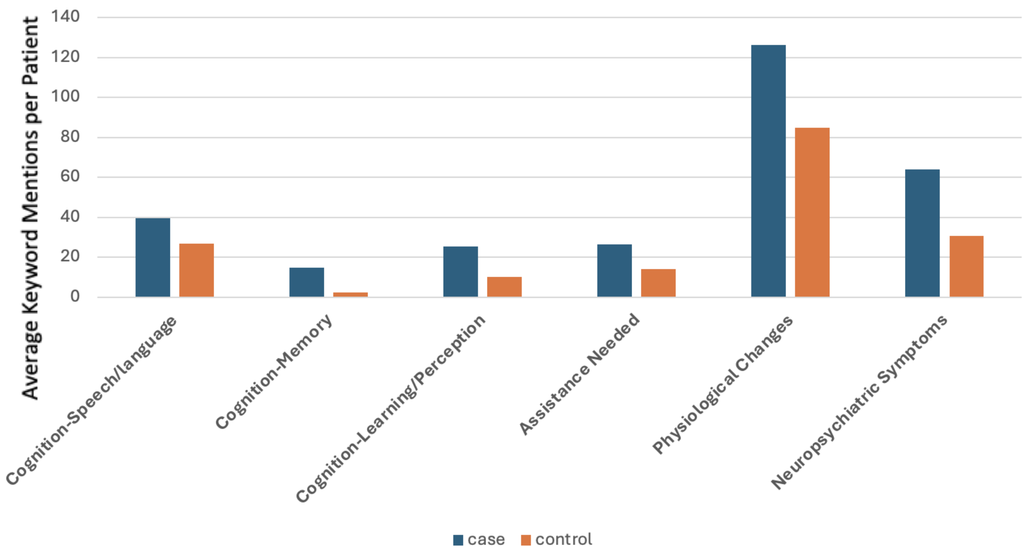

Comparison of average occurrences of subjective cognitive decline (SCD)– and Alzheimer's disease (AD)–related keywords across major symptom domains in AD cases and matched controls. Symptom domains include cognition (speech/language, memory, learning/perception), assistance needed, physiological changes, and neuropsychiatric symptoms.

Supplementary Figure 3. SCD and AD dementia -related keyword patterns in primary care notes.

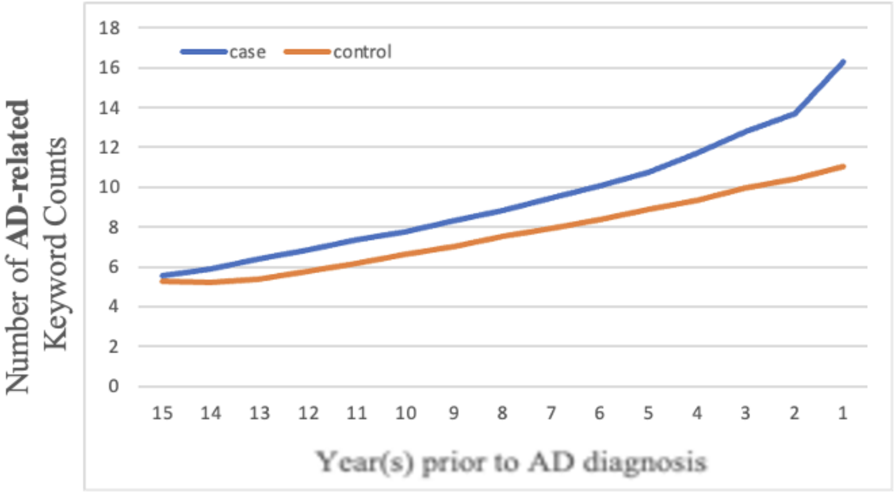

Average number of expert-curated subjective cognitive decline (SCD) and Alzheimer's disease (AD)–related keywords per patient per year in AD cases (n = 61,537) and matched controls (n = 234,105), based on primary care notes from the CP-I high-sensitivity computable phenotype cohort.

Supplementary Figure 4. SCD and AD dementia-related keyword patterns in longitudinal EHRs stratified by age.

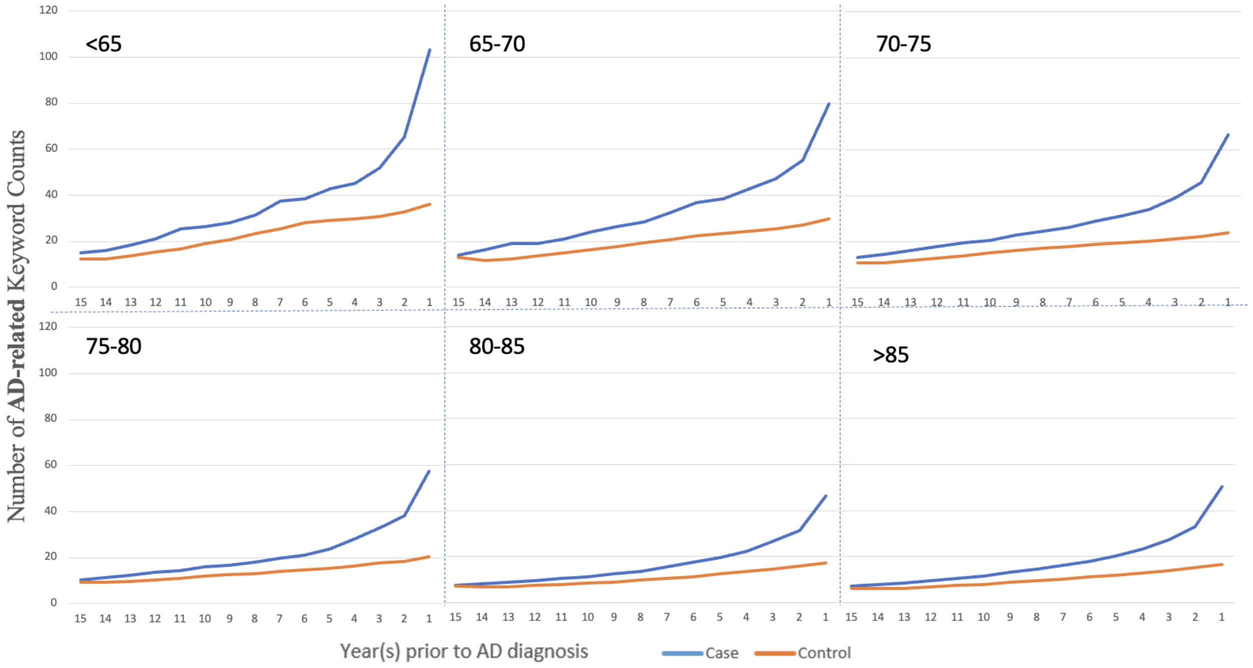

Average annual counts of expert-curated subjective cognitive decline (SCD) and Alzheimer's disease (AD)–related keywords per patient in AD cases and matched controls, stratified by age at diagnosis/index date (<65, 65–70, 70–75, 75–80, 80–85, >85). Data are from the CP-I high-sensitivity computable phenotype cohort (AD cases: 61,537; controls: 234,105).

Supplementary Figure 5. SCD and AD dementia-related keyword patterns in longitudinal EHRs stratified by sex.

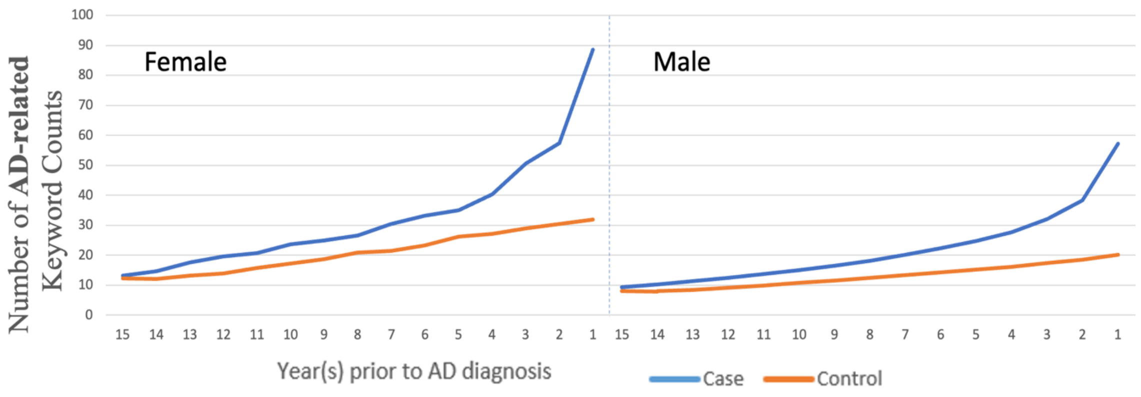

Average annual counts of expert-curated subjective cognitive decline (SCD) and Alzheimer's disease (AD)–related keywords per patient in AD cases and matched controls, stratified by sex (female, male). Data are from the CP-I high-sensitivity computable phenotype cohort (AD cases: 61,537; controls: 234,105).

Supplementary Figure 6. SCD and AD dementia-related keyword patterns in longitudinal EHRs stratified by ethnicity/race.

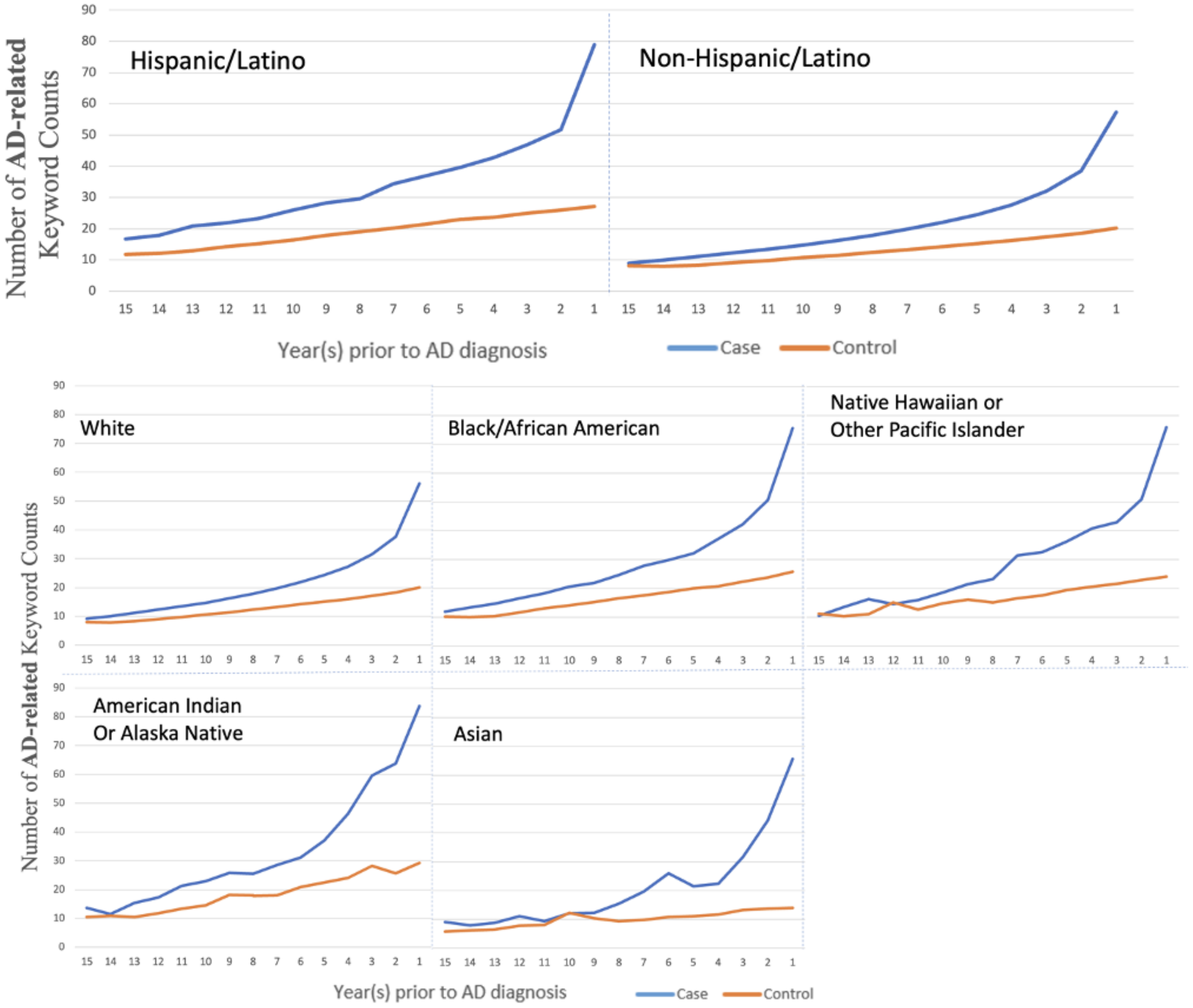

Average annual counts of expert-curated subjective cognitive decline (SCD) and Alzheimer's disease (AD)–related keywords per patient in AD cases and matched controls, stratified by ethnicity (Hispanic/Latino, Non-Hispanic/Latino), race (White, Black/African American, Native Hawaiian or Other Pacific Islander, American Indian or Alaska Native, Asian). Data are from the CP-I high-sensitivity computable phenotype cohort (AD cases: 61,537; controls: 234,105).

Supplementary Figure 7. SCD and AD dementia-related keyword patterns in longitudinal EHRs stratified by symptom domain.

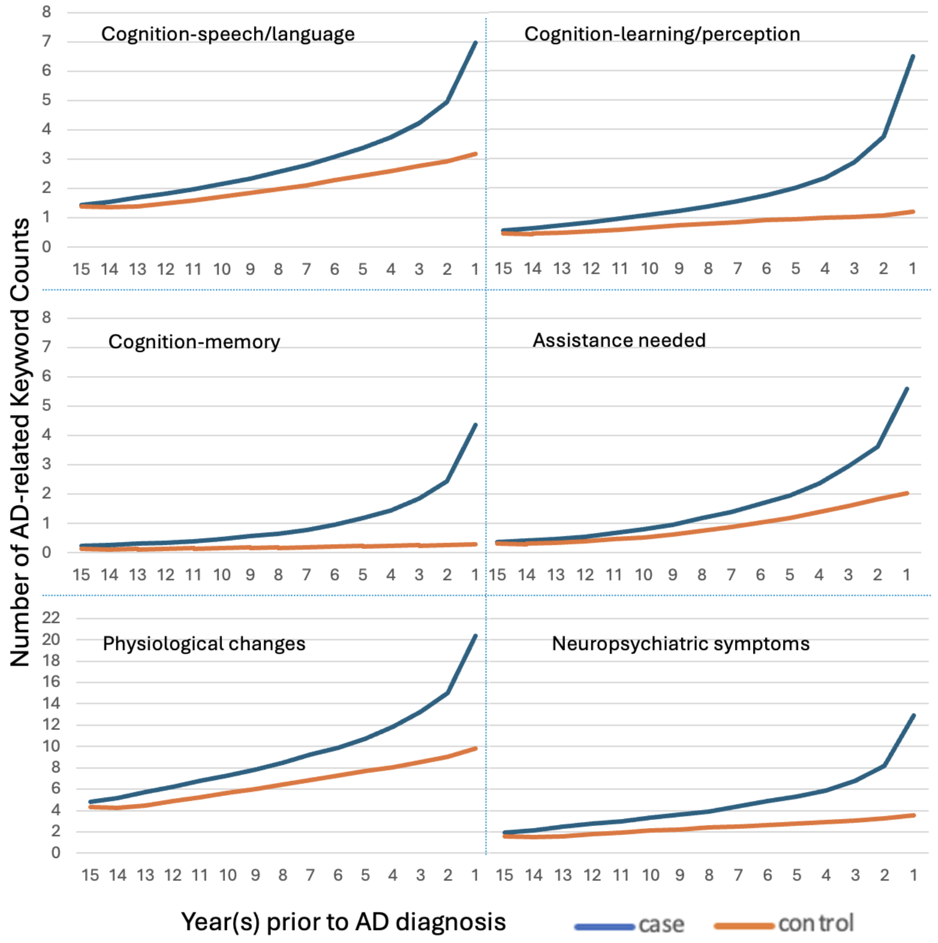

Average annual counts of expert-curated subjective cognitive decline (SCD) and Alzheimer's disease (AD)–related keywords per patient in AD cases and matched controls, stratified by symptom domain (cognition–speech/language, cognition–learning/perception, cognition–memory, assistance needed, physiological changes, neuropsychiatric symptoms). Data are from the CP-I high-sensitivity computable phenotype cohort (AD cases: 61,537; controls: 234,105).

Supplementary Figure 8. SCD and AD dementia-related keyword patterns in longitudinal EHRs in the CP-II cohort.

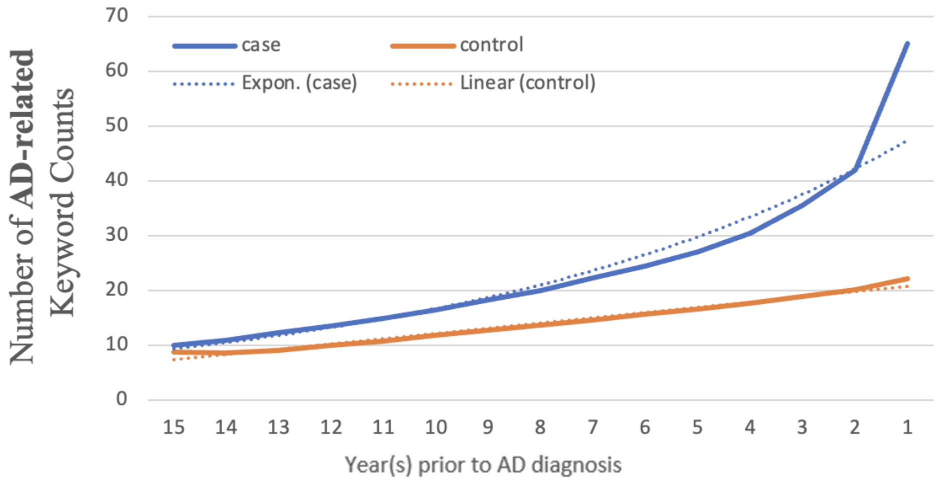

Average counts of SCD and AD dementia-related keywords per patient by year before diagnosis for AD cases and matched controls. Solid lines show observed data, and dotted lines show fitted trendlines (exponential for cases, linear for controls). Data are from the CP-II high-specificity computable phenotype cohort (AD cases: 35,308; controls:145,198). Expert-curated SCD and AD dementia-related keywords were used to identify signs and symptoms. AD, Alzheimer's disease; SCD, subjective cognitive decline.

Supplementary Figure 9. SCD and AD dementia-related keyword patterns in longitudinal EHRs stratified by note types.

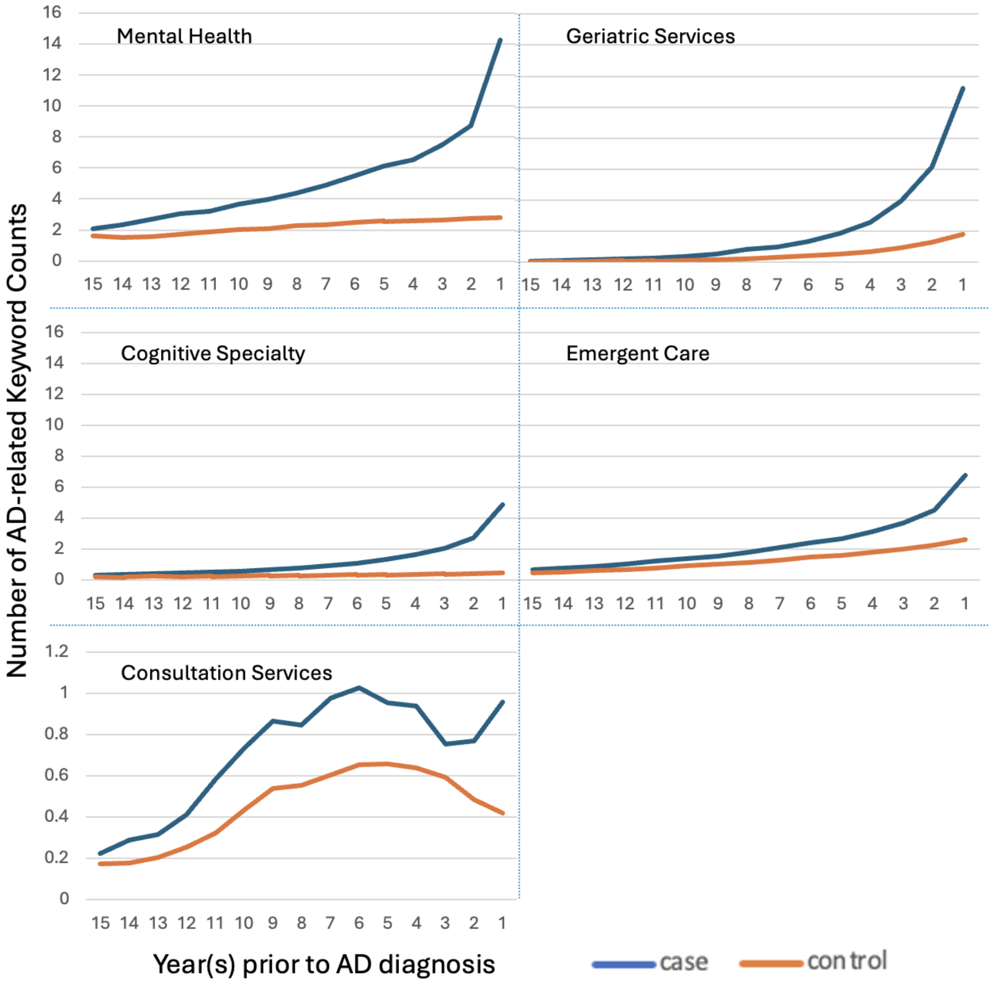

Average counts of subjective cognitive decline (SCD) and Alzheimer's disease (AD)–related keywords per patient per year before diagnosis, stratified by note type. Statistics are based on the CP-I high-sensitivity computable phenotype cohort (AD cases: 61,537; controls: 234,105), using expert-curated keywords to identify signs and symptoms.

Supplementary Figure 10. Distribution of note types by specialty in the case and control cohorts in CP-I cohort.

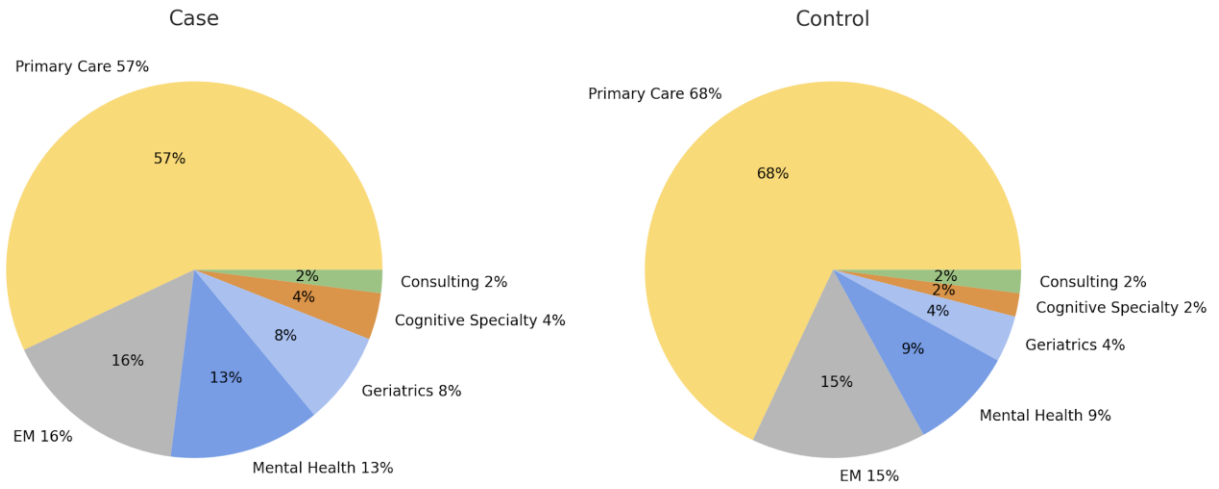

Distribution of note types by specialty in AD cases and matched controls from the CP-I high-sensitivity computable phenotype cohort. Pie charts show the percentage of notes contributed by primary care, emergency medicine (EM), mental health, cognitive specialty, geriatrics, and consulting.

Supplementary Figure 11. SCD and AD dementia–related keyword counts by category over the 3 years preceding diagnosis.

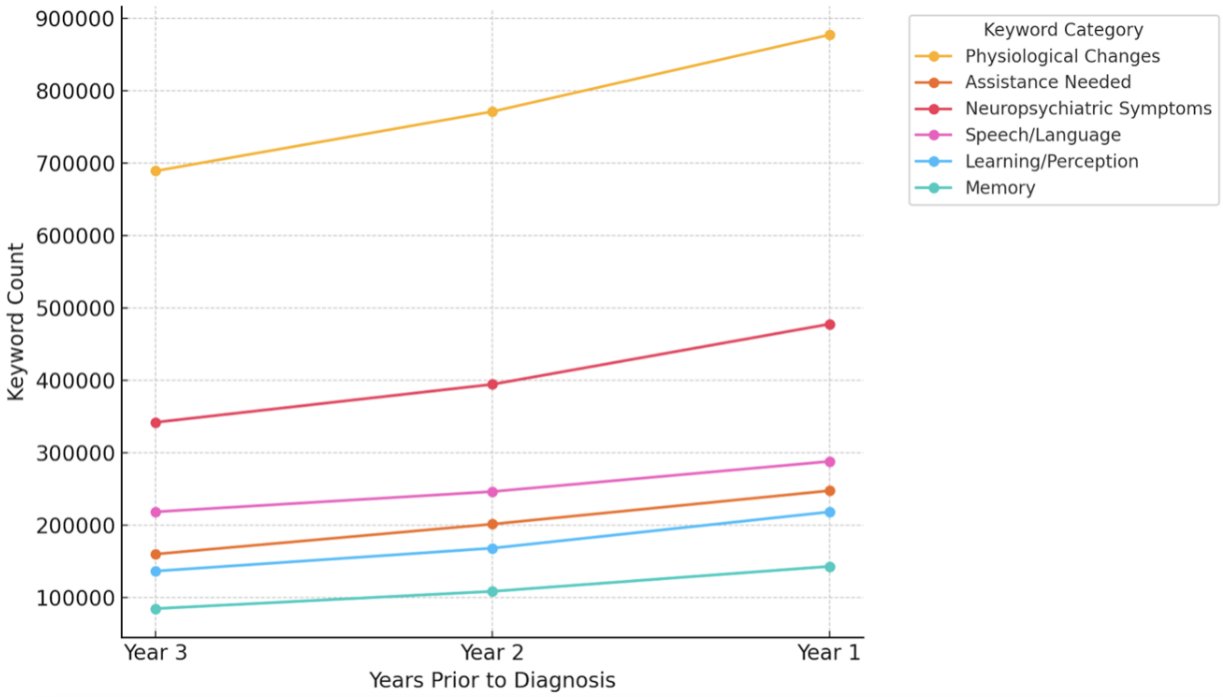

Total counts of subjective cognitive decline (SCD) and Alzheimer's disease (AD)–related keywords by category (physiological changes, assistance needed, neuropsychiatric symptoms, speech/language, learning/perception, memory) over the three years preceding diagnosis. Data are from the CP-I high-sensitivity computable phenotype cohort (AD cases: 61,537; controls: 234,105).

Supplementary Figure 12. Note volume by type in the 3 years preceding diagnosis.

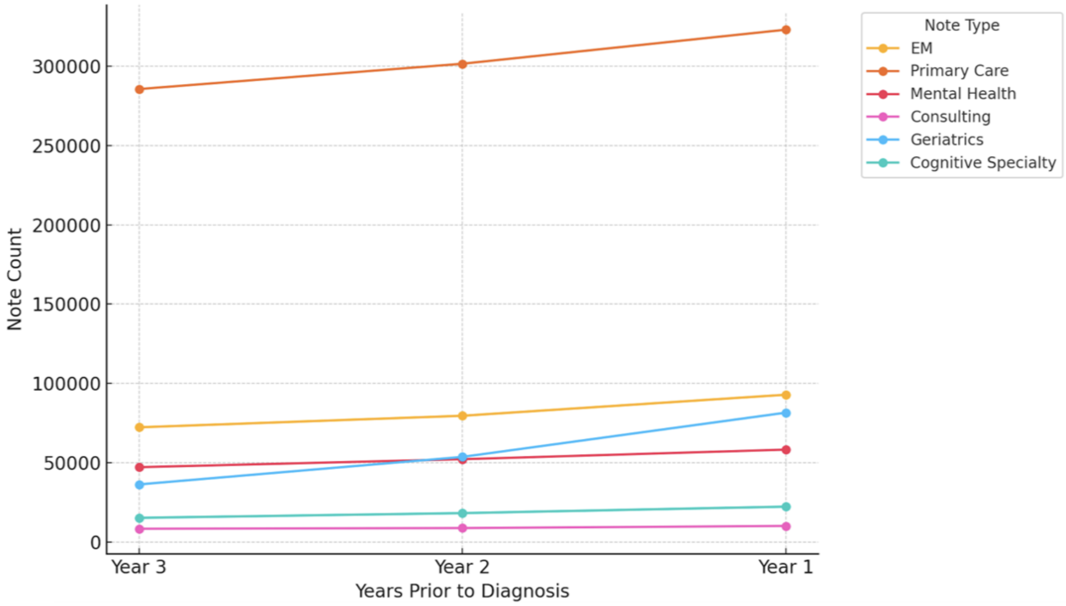

Note volume by type in the three years preceding Alzheimer's disease (AD) diagnosis. Categories include primary care, emergency medicine (EM), mental health, consulting, geriatrics, and cognitive specialty. Data are from the CP-I high-sensitivity computable phenotype cohort (AD cases: 61,537; controls: 234,105).

Supplementary Figure 13. Dermatologic-related keyword patterns in longitudinal EHRs

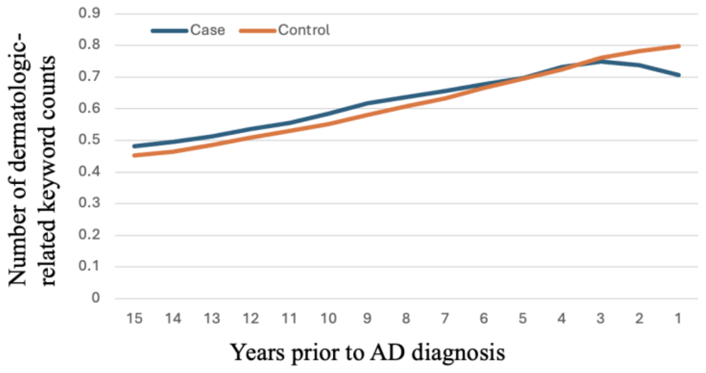

Average number of dermatologic-related keywords per note for Alzheimer's disease (AD) cases and matched controls, plotted by year before diagnosis. Data are from the CP-I high-sensitivity computable phenotype cohort (AD cases: 61,537; controls: 234,105).

Supplementary Figure 14. Accumulated keywords feature group importance ranking of random forest model.

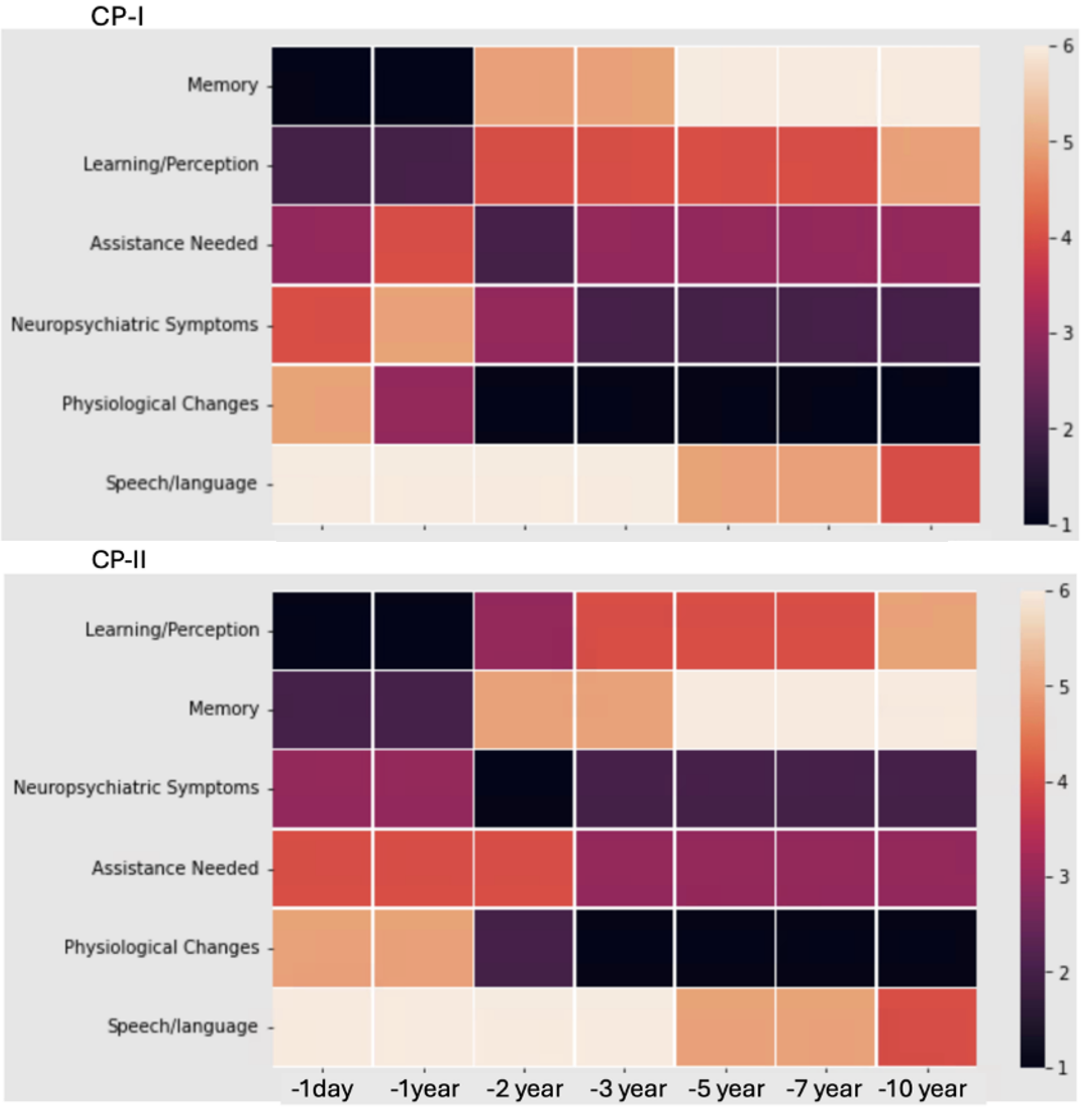

The x-axis depicts the time in days or years prior to the AD diagnosis. The y-axis represents the importance rankings of the features, where smaller numbers (indicated by darker shading) correspond to higher importance/rank.

Supplementary Figure 15. Keywords importance ranking of random forest model on CP-I.

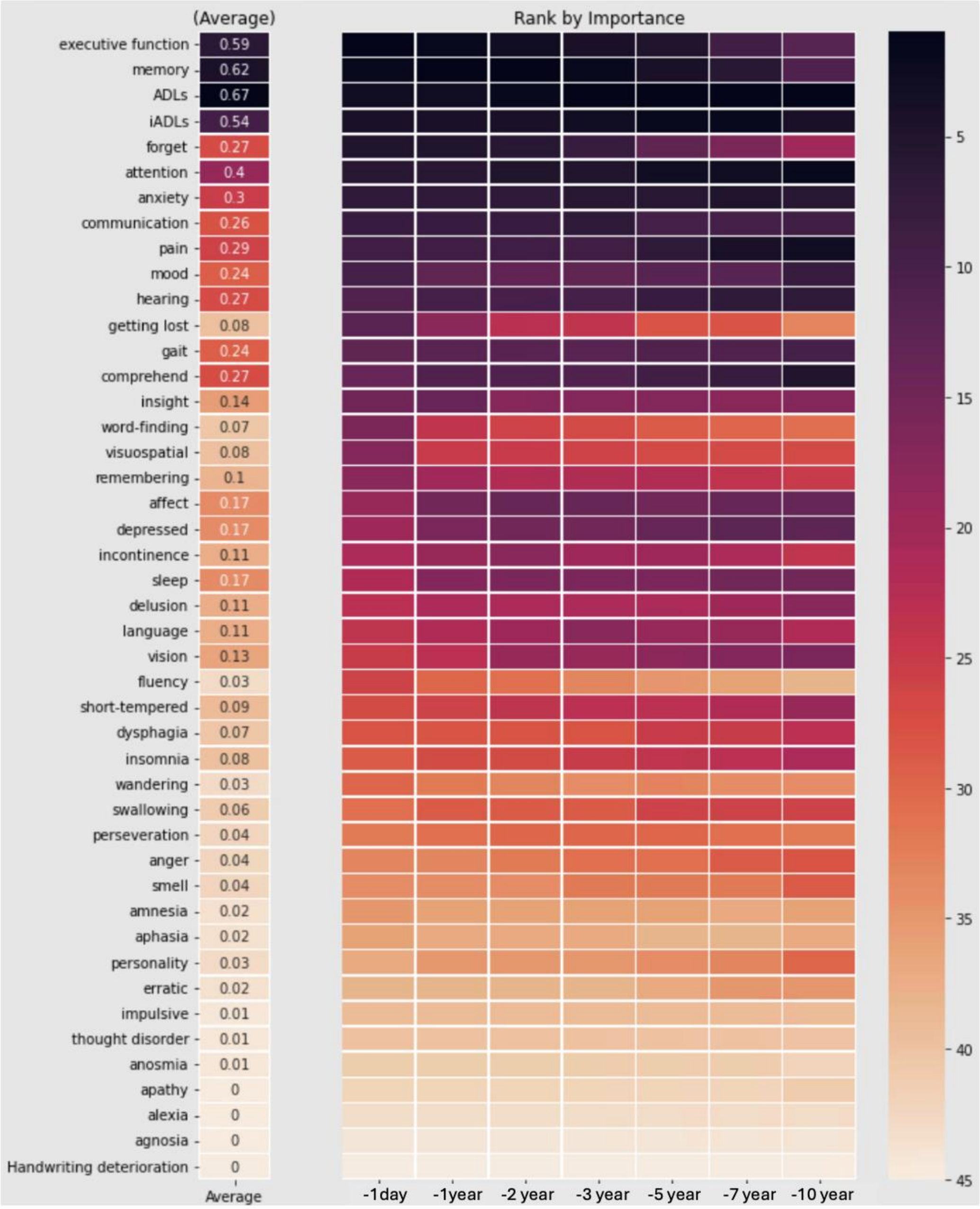

The x-axis depicts the time in days or years prior to the AD diagnosis. The y-axis represents the importance rankings of the features, where smaller numbers (indicated by darker shading) correspond to higher importance/rank.

Supplementary Figure 16. Keywords importance ranking of random forest model on CP-II.

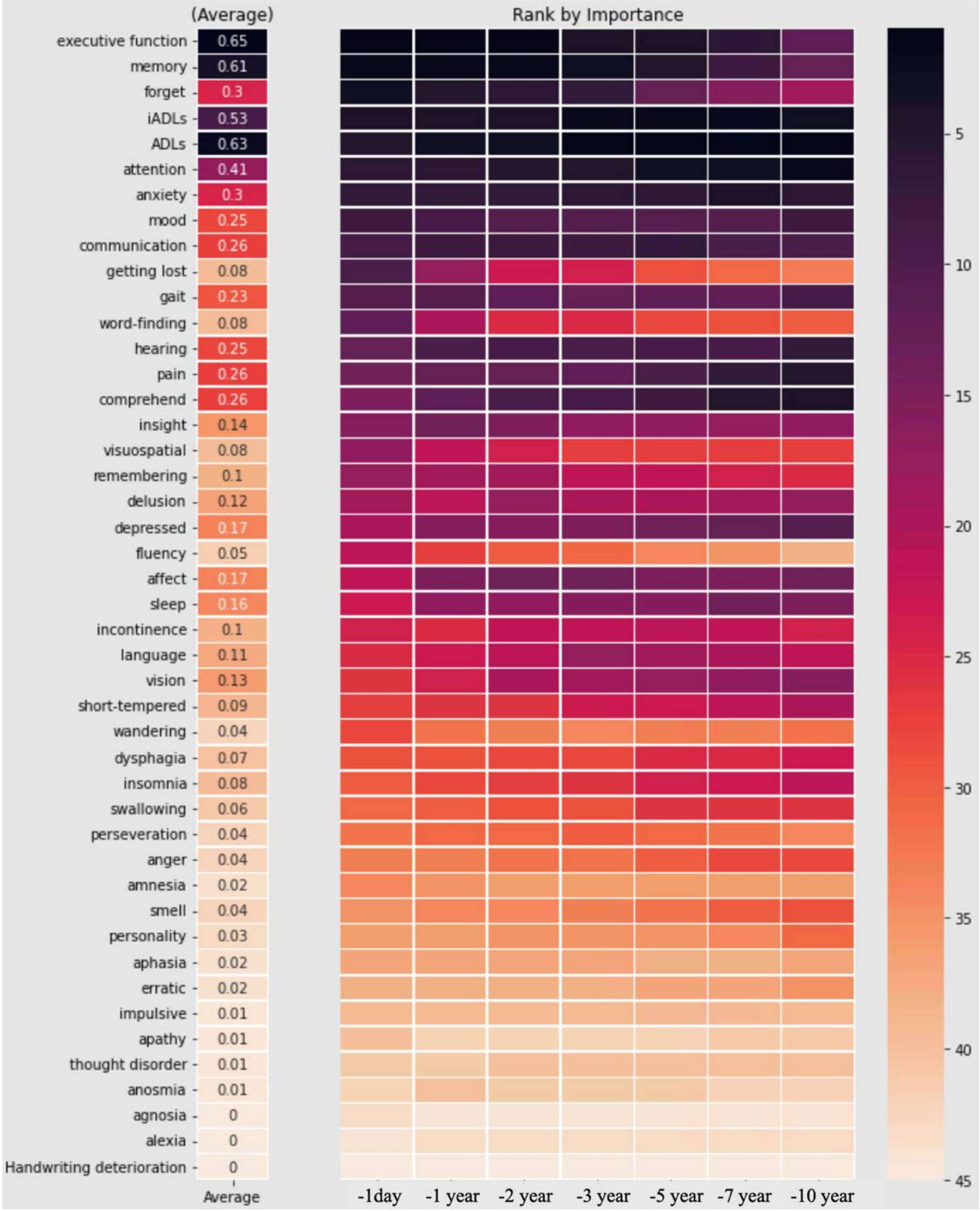

The x-axis depicts the time in days or years prior to the AD diagnosis. The y-axis represents the importance rankings of the features, where smaller numbers (indicated by darker shading) correspond to higher importance/rank.

Supplementary Figure 17. Keywords importance ranking of random forest -1-day model on female/male subgroups.

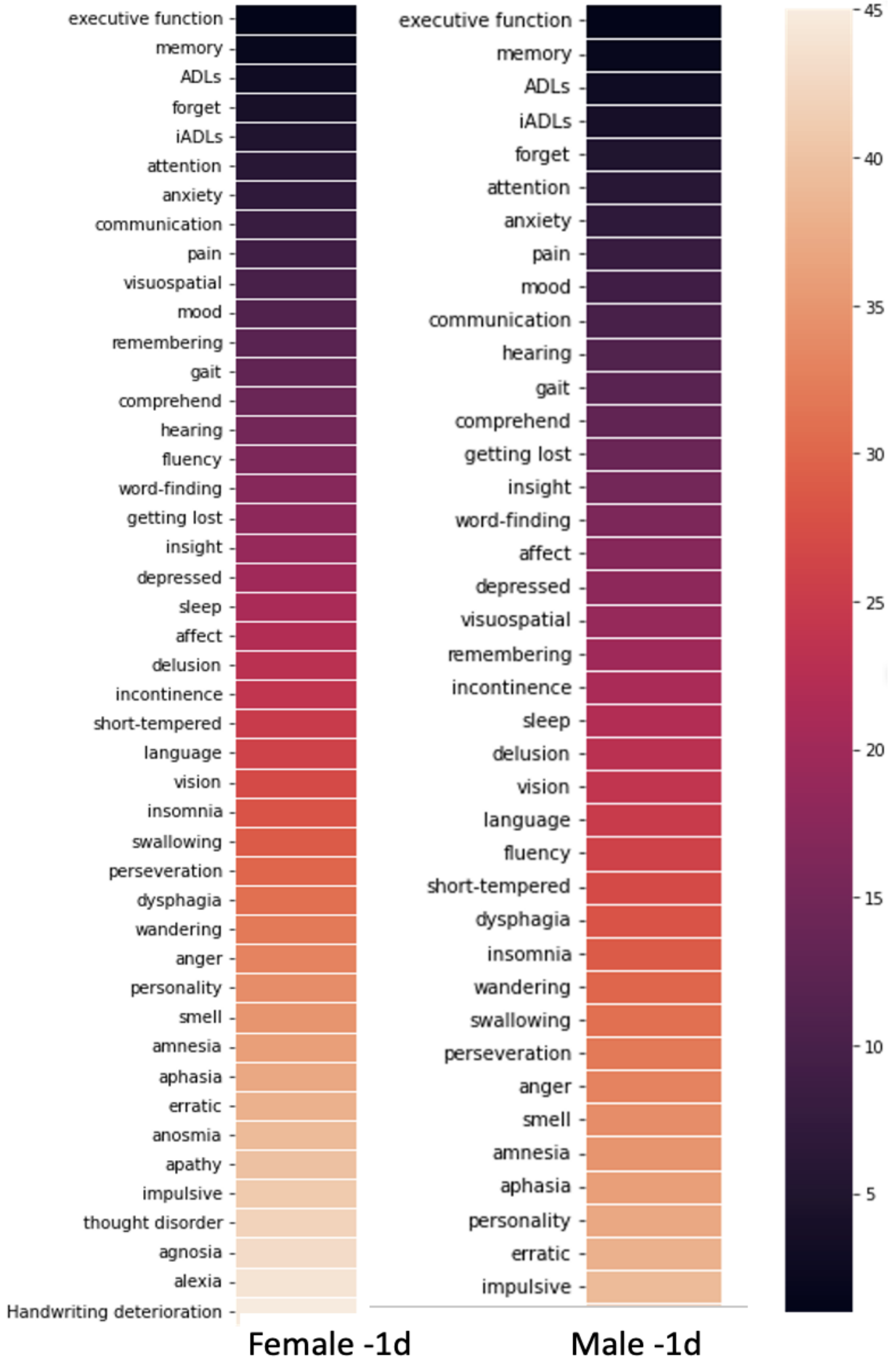

The y-axis represents the importance rankings of the features, where smaller numbers (indicated by darker shading) correspond to higher importance/rank

Supplementary Figure 18. Keywords importance ranking of random forest -1-day model on white and black/African subgroups.

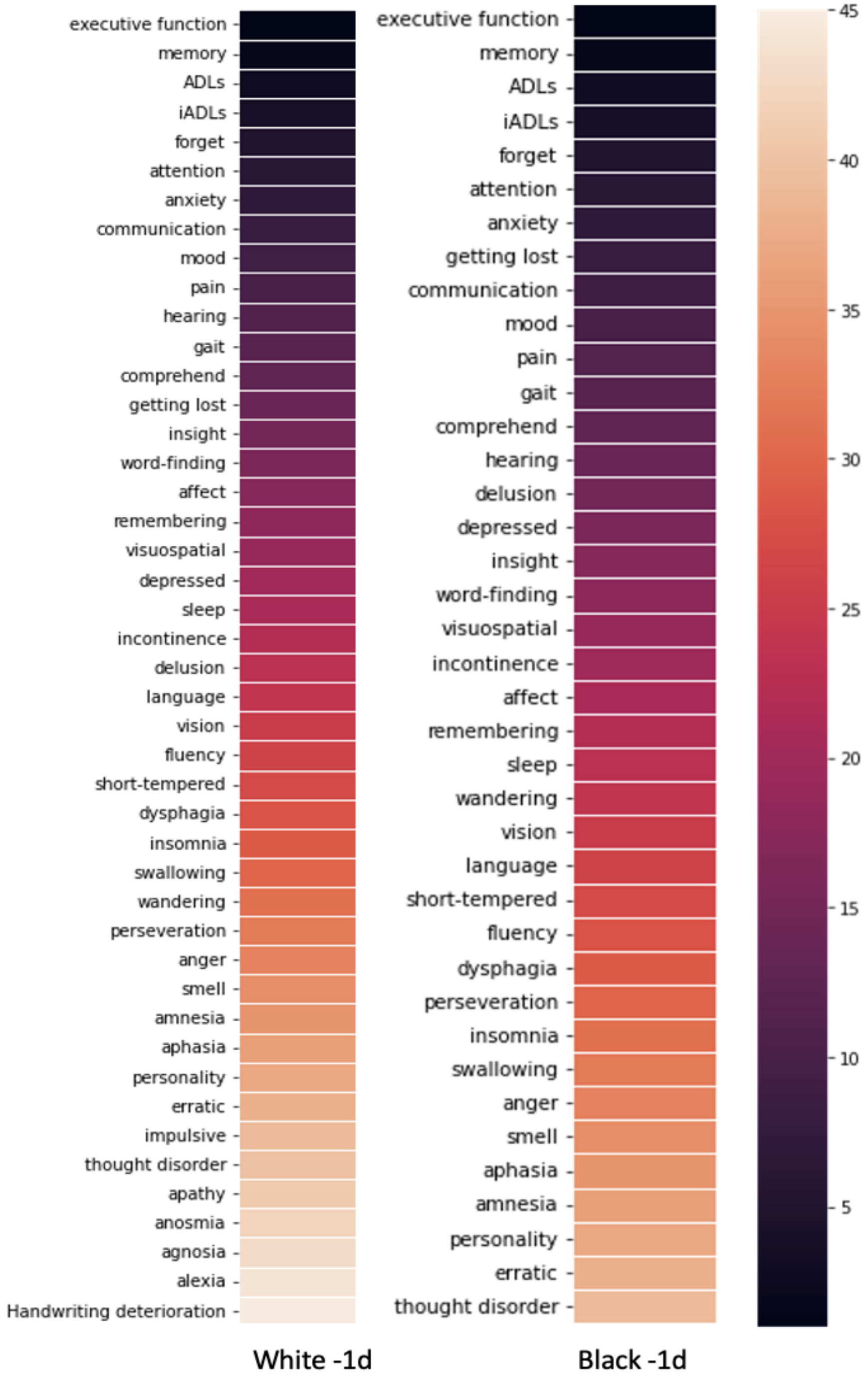

The y-axis represents the importance rankings of the features, where smaller numbers (indicated by darker shading) correspond to higher importance/rank.

Supplementary Figure 19. Keywords importance ranking of random forest -1-day model on Non-Hispanic/Latino and Hispanic/Latino subgroups.

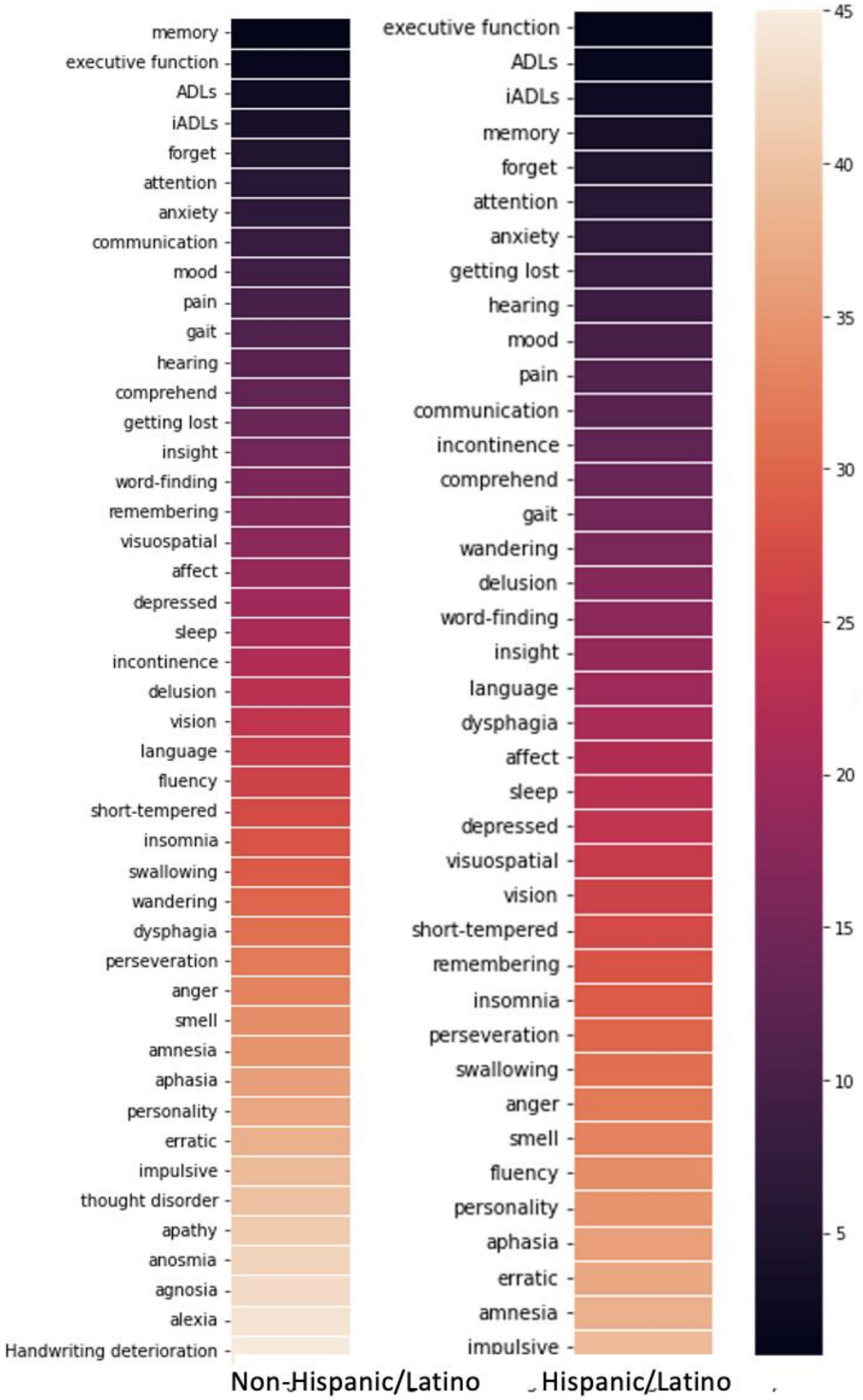

The y-axis represents the importance rankings of the features, where smaller numbers (indicated by darker shading) correspond to higher importance/rank.

Supplementary Table 1. Alzheimer's disease -related ICD code.

ICD Code	Description
331·0 (ICD-9)	Alzheimer's disease
G30.0 (ICD-10)	Alzheimer's disease with early onset
G30.1 (ICD-10)	Alzheimer's disease with late onset
G30.8 (ICD-10)	Other Alzheimer's disease
G30.9 (ICD-10)	Alzheimer's disease, unspecified

ICD, International classification of Diseases.

Supplementary Table 2: ICD Codes used to identify and exclude dementia patients from control groups.

Diagnostic Category	ICD codes
Alzheimer's Disease	331.0, G30.0, G30.1, G30.8, G30.9
Non-Specific Dementia/ Related Dementia/ Other Dementias	294.20, 294.21, F03.90, F03.91: Unspecified dementia without/with behavioral disturbance<br>290.0, 290.20, 290.21, 290.3: Senile dementia variants<br>331.2, G31.1 Senile degeneration of the brain<br>331.6: Corticobasal degeneration<br>331.7: Cerebral degeneration in diseases classified elsewhere<br>331.9: Cerebral degeneration, unspecified<br>331.89: Other cerebral degeneration<br>330.9: Unspecified cerebral degeneration in childhood<br>797: Senility without mention of psychosis<br>G31.9: Degenerative disease of nervous system, unspecified<br>290.40-290.43, F01.50-F01.54: Vascular dementia<br>331.1, 331.19, G31.0, G31.09: Frontotemporal/Other Fronto-Dementia<br>331.82, G31.83: Lewy body dementia<br>290.10, 290.10, 290.11, 290.12, 290.13, G30.0: Presenile/Early onset dementia<br>331.5, G91.2: Idiopathic Normal Pressure Hydrocephalus<br>046.1, A81.00, A81.01, A81.09: Creutzfeldt-jakob disease<br>331.11, G31.01: Pick's disease<br>F10.96 Korsakoff Syndrome<br>294.10, 294.11: Dementia in conditions classified elsewhere with/without behavioral disturbance.<br>F02.80, F02.81, F02.811, F02.818, F02.82, F02.83, F02.84: Dementia in other diseases classified elsewhere with/without behavioral disturbance<br>046.3, A81.2: Progressive multifocal leukoencephalopathy<br>046.79: Other and unspecified prion disease of central nervous system<br>291.1: Alcohol-induced persisting amnestic disorder<br>291.2: Alcohol-induced persisting dementia<br>292.82: Drug-induced persistent dementia<br>292.83: Drug-induced persistent amnestic disorder<br>294.0: Amnestic disorder in conditions classified elsewhere<br>799.55: Frontal lobe and executive function deficit<br>G31.2: Degeneration of nervous system due to alcohol<br>G31.85: Corticobasal degeneration<br>G31.89: Other specified degenerative diseases of nervous system<br>G13.2, G13.8: Systemic atrophy primarily affecting the central nervous system in myxedema<br>G94: Other disorders of brain in diseases classified elsewhere<br>R41.840, R41.841, R41.842, R41.843, R41.844: Other specified cognitive deficit<br>R54: Age-related physical debility<br>F04: Amnestic disorder due to known physiological condition<br>F05: Delirium due to known physiological condition<br>F10.7, F11.7, F12.7, F13.7, F14.7, F15.7, F16.7, F18.7, F19.7, F10.6, F11.6, F12.6, F13.6, F14.6, F15.6, F16.6, F18.6, F19.6: Mental and behavioral disorders<br>A81.89: Other atypical virus infections of central nervous system<br>I67.3: Progressive vascular leukoencephalopathy<br>R41.81: Age-related cognitive decline
Mild Cognitive Impairment (MCI)	331.83, G31.84

ICD, International classification of Diseases.

Supplementary Table 3. Alzheimer's disease-related stop codes within Veterans Health Administration.

Stop Code	Description
320	Dementia clinic (discontinued in Oct 2021)
315	Neurology
318	Geriatric problem-focused consultation clinic (including memory clinic)
350	Geriatric primary care
538	Psychological testing
502	Mental health clinic
576	Psycho-geriatric clinic
509	Psychiatry

Supplementary Table 4: Logistic regression and XGBoost prediction results using keyword features in Setting I on the CP-I cohort.

	Logistic Regression		XGBoost	
Prediction Horizon	AUROC	AUPRC	AUROC	AUPRC
-1 day	0.826	0.670	0.856	0.732
-1 year	0.745	0.545	0.770	0.598
-2 year	0.707	0.483	0.731	0.532
-3 year	0.679	0.438	0.703	0.484
-5 year	0.636	0.369	0.657	0.406
-7 year	0.607	0.331	0.624	0.361
-10 year	0.570	0.356	0.580	0.371

AUROC, Area Under the Receiver Operating Characteristic Curve; AUPRC, Area Under the Precision–Recall Curve; CP-I, high-sensitivity computable phenotype.

Supplementary Table 5. Random forest prediction results using keyword features from different specialty note types in Setting I on the CP-I cohort.

	Emergent Care		Mental Health		Cognitive Specialty		Consultation Services		Geriatric Service	
Prediction Horizon	AUROC	AUROC	AUPRC	AUPRC	AUPRC	AUPRC	AUPRC	AUPRC	AUPRC	AUPRC
-1 day	0.618	0.384	0.648	0.455	0.642	0.447	0.561	0.311	0.603	0.385
-1 year	0.585	0.33	0.603	0.381	0.595	0.371	0.546	0.28	0.555	0.309
-2 year	0.567	0.305	0.583	0.35	0.575	0.338	0.539	0.265	0.539	0.283
-3 year	0.557	0.289	0.572	0.325	0.558	0.312	0.537	0.258	0.529	0.265
-5 year	0.547	0.269	0.549	0.289	0.538	0.278	0.527	0.248	0.517	0.247
-7 year	0.535	0.26	0.536	0.272	0.525	0.261	0.519	0.241	0.513	0.242
-10 year	0.519	0.304	0.522	0.314	0.511	0.301	0.510	0.236	0.504	0.292

AUROC, Area Under the Receiver Operating Characteristic Curve; AUPRC, Area Under the Precision–Recall Curve; CP-I, high-sensitivity computable phenotype.
