# Supplementary material for: Early prediction of Alzheimer’s disease using longitudinal electronic health records of US military veterans
Source: Commun Med (Lond). 2026 Jan 12;6:23. doi: 10.1038/s43856-025-01206-w (PMC12796311; doi:10.1038/s43856-025-01206-w)
Supplement: Supplementary file 3 — Description of Additional Supplementary Data [file 43856_2025_1206_MOESM3_ESM.pdf]

Description of additional supplementary file

File name: Supplementary Data 1

Description: source data for Figures 3 and 4
